# Effects of floral resources on honey bee populations in Mexico: Using dietary metabarcoding to examine landscape quality in agroecosystems

**DOI:** 10.1002/ece3.11456

**Published:** 2024-06-17

**Authors:** Francisco J. Balvino‐Olvera, Ulises Olivares‐Pinto, Antonio González‐Rodríguez, María J. Aguilar‐Aguilar, Gloria Ruiz‐Guzmán, Jorge Lobo‐Segura, Jorge Cortés‐Flores, E. Jacob Cristobal‐Perez, Silvana Martén‐Rodríguez, Violeta Patiño‐Conde, Mauricio Quesada

**Affiliations:** ^1^ Laboratorio Nacional de Análisis y Síntesis Ecológica, Escuela Nacional de Estudios Superiores Unidad Morelia Morelia Michoacán Mexico; ^2^ Posgrado en Ciencias Biológicas, Unidad de Posgrado, Edificio D, 1° Piso, Circuito de Posgrados Ciudad Universitaria CDMX Mexico; ^3^ Escuela Nacional de Estudios Superiores Unidad Juriquilla Universidad Nacional Autónoma de México Juriquilla Querétaro Mexico; ^4^ Instituto de Investigaciones en Ecosistemas y Sustentabilidad Universidad Nacional Autónoma de México Morelia Michoacán Mexico; ^5^ Escuela de Biología Universidad de Costa Rica San Pedro Costa Rica; ^6^ Laboratorio Binacional de Análisis y Síntesis Ecológica, Escuela de Biología Universidad de Costa Rica San Pedro Costa Rica; ^7^ Jardín Botánico, Instituto de Biología, Sede Tlaxcala Universidad Nacional Autónoma de México Santa Cruz Tlaxcala Mexico

**Keywords:** agriculture intensification, colony losses, floral resources, honey bees, pollen metabarcoding, urbanization

## Abstract

The decline of honey bee populations significantly impacts the human food supply due to poor pollination and yield decreases of essential crop species. Given the reduction of pollinators, research into critical landscape components, such as floral resource availability and land use change, might provide valuable information about the nutritional status and health of honey bee colonies. To address this issue, we examine the effects of landscape factors like agricultural area, urban area, and climatic factors, including maximum temperature, minimum temperature, relative humidity, and precipitation, on honey bee hive populations and nutritional health of 326 honey bee colonies across varying landscapes in Mexico. DNA metabarcoding facilitated the precise identification of pollen from 267 plant species, encompassing 243 genera and 80 families, revealing a primary herb‐based diet. Areas characterized by high landscape diversity exhibited greater pollen diversity within the colony. Conversely, colonies situated in regions with higher proportions of agricultural and urban landscapes demonstrated lower bee density. The maximum ambient temperature outside hives positively correlated with pollen diversity, aligning with a simultaneous decrease in bee density. Conversely, higher relative humidity positively influenced both the bee density of the colony and the diversity of foraged pollen. Our national‐level study investigated pollen dietary availability and colony size in different habitat types, latitudes, climatic conditions, and varied levels and types of disturbances. This effort was taken to gain a better insight into the mechanisms driving declines in honey bee populations. This study illustrates the need for more biodiverse agricultural landscapes, the preservation of diverse habitats, and the conservation of natural and semi‐natural spaces. These measures can help to improve the habitat quality of other bee species, as well as restore essential ecosystem processes, such as pollination and pest control.

## Graphical abstract

This study investigates how landscape diversity and climatic factors affect the populations and nutritional health of honey bee colonies in Mexico. It was found that greater landscape diversity correlates with higher pollen diversity, while areas with more agriculture and urbanization show lower bee densities. The results underscore the importance of preserving diverse habitats to support bee health and, by extension, human food security.

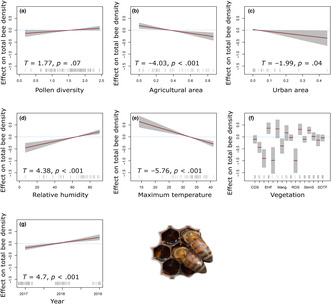

## 1 INTRODUCTION

Floral diversity, abundance, and nutritional content are related to colony health, as pollen and nectar provide all the carbohydrates and micro and macro‐nutrients required for the honey bee colony survival (Trinkl et al., 2020). Honey bees (*Apis mellifera* L.) that consume pollen with high quality and diversity have a better life expectancy and immunity and are more tolerant to parasites and pathogens (Dolezal & Toth, 2018; Pasquale et al., 2013). Honey bees are eusocial species that collect floral resources using a complex communication system within the colony (Seeley, 2019). Specifically, honey bee foragers communicate the location of food resources to their siblings using a characteristic waggle dance (Frisch & Seeley, 1967). The highly specialized communication systems, along with another simpler set, enable these organisms to engage in foraging behavior effectively. This adaptive capability allows them to continually adjust to changing landscape conditions, thereby optimizing their ability to make the most efficient use of available resources (Seeley, 2019). Moreover, honey bees can balance their nutritional intake by foraging from a diversity of complementary food sources, thus achieving optimal nutrition (Arien et al., 2015, 2020; Ruedenauer et al., 2021). Floras in the tropics offer diverse diets to honey bees due to high species diversity and high floral resources availability throughout the year, related to staggered flowering phenologies (Alaux et al., 2017; Pasquale et al., 2016; Smart et al., 2019). However, the loss of natural and semi‐natural areas implies a reduction in the availability and quality of floral resources for bees (Baude et al., 2016), with concomitant negative impacts on bee health and behavior (Brodschneider et al., 2018; Dolezal & Toth, 2018; McNeil et al., 2020; Vaudo et al., 2015). Land use change is considered the primary cause of bee decline (Goulson et al., 2015; Hristov et al., 2021; Shanahan, 2022; Winfree et al., 2009).

The European honey bee is a generalist pollinator that is highly important for crop pollination (Klein et al., 2007), particularly in tropical countries such as Mexico, where animal pollination is required for fruit and seed production of 85% of edible plants (145 crop species; Ashworth et al., 2009). A quantifiable economic value of almost USD 2 billion in 2009 highlights the pollinator's indispensable contribution to this country (Ashworth et al., 2009). Moreover, the honey bee is an essential pollinator in natural ecosystems, playing a preponderant role in the sexual reproduction, gene flow, and genetic diversity maintenance of a large number of plant species (Cortés‐Flores et al., 2023; Delgado‐Carrillo et al., 2018; Dick, 2001). Their significance extends beyond mere pollination services for food production, encompassing areas such as honey, wax, and royal jelly production, with significant economic implications (Degrandi‐Hoffman et al., 2019). In Mexico, beekeeping holds social significance, particularly for small‐scale beekeepers, who constitute approximately 70% of the industry (Balvino‐Olvera et al., 2023). These beekeepers heavily rely on the sale of honey and bee by‐products as a significant source of income, further emphasizing the multi‐faceted importance of honey bees in the economy and society (Arechavaleta‐Velasco et al., 2021). However, trends of declining bee populations around the world raise concerns about implications for plant reproduction, food security, and broader economic consequences (Balvino‐Olvera et al., 2023; Mashilingi et al., 2022).

Mexico stands as a living laboratory, rich in a variety of landscapes that support numerous types of vegetation, as well as a wide range of climates and ecosystems. A recent study using long‐term data from beekeeping across Mexico revealed a significant correlation between climatic conditions, agricultural land use, and honey bee colony trends. Increased industrial agriculture and higher temperatures resulted in reduced honey yield, while climate change‐induced decreases in temperature ranges led to a decline in the number of hives (Balvino‐Olvera et al., 2023). To expand on this knowledge at the landscape level and field conditions, in this study, we analyzed commercial honey bee hives, examining critical landscape components, such as the effects of land use change and environmental conditions on pollen diversity and honey bee colony size. Previous research on colony losses in agroecosystems has highlighted the importance of monitoring the status of population growth, with a particular emphasis on brood and adult populations. These metrics are crucial indicators of colony production, fitness, and lifespan (Alburaki, Cheaib, et al., 2017; Ricigliano et al., 2019). On the other hand, pollen taxonomical diversity in bee hives can be used to extrapolate measures of floral community diversity at the landscape level, providing insight into the spatial and temporal dynamics of the nutritional landscape (Millard et al., 2021).

Traditional methods for studying bee foraging diets including direct observation of insect visits to flowers, decoding of bee dances to estimate foraging distances, marking and recapturing of foraging workers, and the use of light microscopy to identify pollen returned to the hive, might have some limitations (Seeley et al., 1991; Sponsler et al., 2017; Tuell et al., 2008; Wood et al., 2018). DNA metabarcoding techniques emerged as promising tools for understanding pollination ecology by increasing plant taxonomic resolution and minimizing time and costs (Laha et al., 2017; Smart et al., 2017). The use of DNA metabarcoding to study the interplay between agricultural practices, pollinator health, and ecosystem biodiversity has become a subject of intense scientific inquiry. Peters et al. (2022) and Trinkl et al. (2020) have shed light on the intricate relationship between resource diversity, nutritional composition, and the well‐being of pollinators by employing a combination of pollen DNA metabarcoding and chemical analyses (Peters et al., 2022; Trinkl et al., 2020). This innovative approach has unveiled the significant repercussions of reduced resource diversity in intensive agricultural landscapes on the nutritional profiles of pollinators. Previous research has established correlations between plant species richness and colony fitness, reproduction, and pollen resource diversity (Alburaki, Steckel, et al., 2017; Ricigliano et al., 2019; Sponsler & Johnson, 2015), but still, critical gaps in understanding persist. Specifically, pollinator nutrition, foraging behavior, and their interaction with changing landscapes in biodiverse regions, in the face of land change use and climatic changes, still need to be more adequately explored. We hypothesize that colonies in landscapes with a higher proportion of agricultural land use or urban proportions may experience weakened colony strength due to a lack of access to high‐diversity floral resources and poor nutrition. We examine the effects of landscape factors associated with agricultural and urban areas and climatic factors including maximum temperature, minimum temperature, relative humidity, and precipitation, on honey bee hive population size and pollen diversity. Our research seeks to provide insights into the decline of honey bee populations in Mexico, offering valuable information for conservation policies in agrosystem management.

## 2 MATERIALS AND METHODS

### 2.1 Study region and honey bee hive size

**FIGURE 1:**
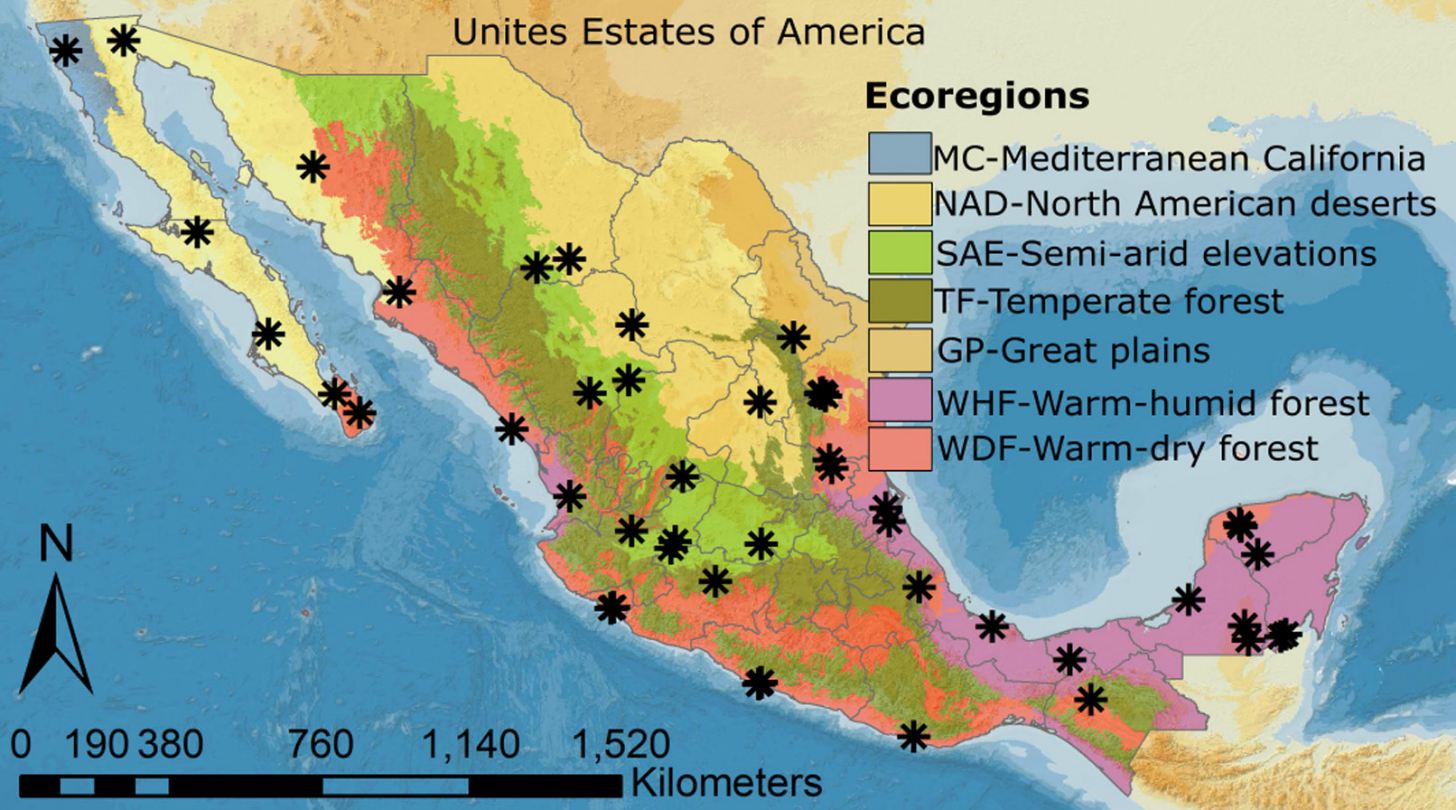
Map depicting the geographic location of apiaries sampled in Mexico. Colors indicate the level I ecoregions.

Fifty‐two apiaries were chosen from Mexico ecoregions with the assistance and collaborative work of the Beekeeping Product System Association of México (Sistema Producto Apícola de México) (Figure 1). To accomplish our objectives, we assessed colony strength across two seasons, and we chose colonies with comparable bee activity at the hive entrance and an identical number of honey supers. After consulting with the involved beekeepers, we have decided to keep the selected colonies on‐site for as long as possible to enable re‐sampling in the subsequent season. This approach has enabled us to assess the ongoing development and conditions of the colonies within a more controlled and consistent environment (Delaplane et al., 2013). Based on historical daily precipitation averages for each site (EMA's, 2022), the sampling season for Mexico was determined due to the highly variable seasonal precipitation patterns. Between 2017 and 2019, three to four hives per apiary were sampled in each season, resulting in a total of 326 hives collected. Specifically, 186 hives were collected during the rainy season and 140 hives during the dry season (Table 1). An analysis of the maternal and paternal ancestry in these colonies shows that honey bee colonies are mainly Africanized, resulting from a mix of *Apis mellifera scutellata* from Africa and subspecies from Europe. The proportion of African ancestry varies depending on factors such as management practices, beekeeping locations, and latitude (Aguilar‐Aguilar et al., 2024). An inspection of the colony was performed on each selected colony, which involved removing each frame from the brood chamber to identify the presence of eggs, larvae, capped brood, nursing bees, diseases, and other organisms, as well as food reserves (pollen and honey). To assess colony strength, we employ a modified version of the Liebefeld method (Dainat et al., 2020; Imdorf, 1987). This approach entails estimating the number of adult workers and brood on each side of the comb and determining the surface area (measured in cm^2^) occupied by brood and workers. As part of this process, two frames were randomly selected from the breeding area (instead of all frames), which was identified during the colony inspection. The breeding area may not be located centrally within the colony due to temperature variations influenced by the hive's orientation and volume (Seeley, 1977; Seeley & Morse, 1976, 1978).

**TABLE 1:** List of apiaries sampled with their respective year, season of collection, vegetation, and ecoregion.

Year	Season	N. of hives	Site	State	Vegetation	Ecoregion
2017	Wet	5	Alamo	Veracruz	EHF	WHF
2018	Dry	4	Alamo	Veracruz	EHF	WHF
2017	Wet	3	Alvaro Obregon	Quintana Roo	SbeMF	WHF
2018	Dry	3	Alvaro Obregon	Quintana Roo	SbeMF	WHF
2017	Wet	6	Buena Vista	San Luis Potosi	SDTF	WDF
2018	Dry	5	Buena Vista	San Luis Potosi	SDTF	WDF
2017	Wet	5	Canon del Sumidero	Chiapas	SDTF	WDF
2018	Dry	4	Cañon del Sumidero	Chiapas	SDTF	WDF
2018	Wet	5	Carbonero	Tamaulipas	SbmS	GP
2017	Wet	5	Centella Km 22	San Luis Potosi	SDTF	WDF
2018	Dry	5	Centella Km 22	San Luis Potosi	SDTF	WDF
2019	Dry	5	Colonia Lerma	B.C.	HV	NAD
2019	Wet	5	Ejido el Aguacate, Tepic	Nayarit	POF	TF
2019	Wet	5	Ejido San Pedro, la Paz	B.C.S.	SDS	NAD
2019	Wet	4	El Botadero, Marismas	Sinaloa	Mang	WHF
2019	Wet	5	El Fuerte	Sinaloa	SDS	WDF
2018	Wet	5	El Jobero I	Guerrero	SDTF	WDF
2018	Wet	4	El Jobero II	Guerrero	SDTF	WDF
2019	Dry	3	El Jobero II	Guerrero	SDTF	WDF
2017	Wet	4	El Lechugal, Alvaro Obregon	Quintana Roo	SbeMF	WHF
2018	Dry	4	El Lechugal, Alvaro Obregon	Quintana Roo	SbeMF	WHF
2017	Wet	4	Gallinero, Merida	Yucatan	SDTF	WDF
2018	Dry	3	Gallinero, Merida	Yucatan	SDTF	WDF
2019	Dry	3	Gustavo Diaz Ordaz, El Vizcaino	B.C.S.	MDS	NAD
2017	Wet	7	Independencia, Teocelo	Veracruz	CF	TF
2018	Dry	2	Independencia, Teocelo	Veracruz	CF	TF
2018	Wet	5	Jamay	Jalisco	SbS	SAE
2019	Dry	4	La Providencia Jamay	Jalisco	SbS	SAE
2017	Dry	12	Jesus Maria	Aguascalientes	SbS	SAE
2017	Wet	4	Jesus Maria	Aguascalientes	SbS	SAE
2017	Wet	3	Kab Kanche	Yucatan	SbdMF	WDF
2019	Dry	5	La Colmena, Hermosillo	Sonora	SDS	NAD
2017	Wet	6	La Mesa de Capula	Guanajuato	CDS	SAE
2017	Wet	3	La Mordida, Cd Victoria	Tamaulipas	SbmS	GP
2017	Dry	10	La Primavera	Jalisco	POF	TF
2017	Wet	4	La Primavera	Jalisco	POF	TF
2017	Wet	5	Las Piletas	Campeche	SbeMF	WHF
2018	Dry	4	Las Piletas	Campeche	SbeMF	WHF
2019	Dry	3	Las Pitahayas, Insurgentes	B.C.S.	SDS	NAD
2018	Wet	5	Las Pozas, Armeria	Colima	SDTF	WDF
2019	Dry	4	Las Pozas, Armeria	Colima	SDTF	WDF
2019	Wet	4	Lopez Rayon	Durango	CDS	SAE
2019	Wet	3	Los Amparan	Chihuahua	Grass	SAE
2017	Wet	6	Los sabinos, Montemorelos	Nuevo Leon	SbmS	GP
2018	Dry	5	Los Sabinos, Montemorelos	Nuevo Leon	SbmS	GP
2017	Wet	4	Marcelino Inurreta	Tabasco	EHF	WHF
2018	Dry	5	Marcelino Inurreta	Tabasco	EHF	WHF
2018	Wet	4	Mariposa‐Carboneros	Tamaulipas	SbmS	GP
2018	Wet	11	Tierra Nueva	Tamaulipas	SbmS	GP
2018	Wet	3	Matehuala	San Luis Potosi	RDS	NAD
2019	Wet	5	Ojo de agua, Jimenez	Chihuahua	MDS	NAD
2017	Wet	3	Papelillo	Campeche	SbeMF	WHF
2018	Dry	7	Papelillo	Campeche	SbeMF	WHF
2019	Dry	4	Piedras gordas, Ensenada	B.C.	Chap	MC
2019	Dry	5	Rancho Cuquito, Santiago	B.C.S.	SDS	NAD
2017	Wet	6	Reforma Agraria	Campeche	SbdMF	WHF
2017	Dry	10	San Carlos	Durango	RDS	NAD
2017	Wet	2	San Carlos	Durango	RDS	NAD
2018	Wet	5	San Fernando, Armeria	Colima	SDTF	WDF
2019	Dry	4	San Fernando, Armeria	Colima	SDTF	WDF
2017	Wet	4	San Jose Tzal, Merida	Yucatan	SDTF	WDF
2018	Dry	4	San Jose Tzal, Merida	Yucatan	SDTF	WDF
2018	Wet	6	San Jose, Patzcuaro	Michoacan	POF	TF
2018	Dry	4	Teocelo	Veracruz	CF	TF
2018	Wet	6	Tetitlan	Guerrero	Mang	WDF
2019	Dry	4	Tetitlan	Guerrero	Mang	WDF

*Note*: Ecoregions (MC = Mediterranean California, NAD = North American Deserts, SAE = Semiarid Elevations, TF = Temperate Forest, GP = Great Plains, WHF = Warm‐humid Forest, WDF = Warm‐dry Forest), vegetation (SbdMF = Sub‐deciduous Medium Forest, SbS = Subtropical Scrub, SbmS = Submontane Scrub, EHF = Evergreen High Forest, CDS = Crassicaule Desert Scrub, SDTF = Seasonally Dry Tropical Forest, Mang = Mangrove, SbeMF = Sub‐evergreen Medium Forest, SDS = Sarcocaule Desert Scrub, MDS = Microphyll Desert Scrub, HV = Halophilic vegetation, CF = Cloud Forest, Chap = Chaparral, RDS = Rosetophyllous Desert Scrub, POF = Pine‐Oak Forest, Grass = Grassland).

The parameter used to measure colony population size was total bee density (worker density + immature worker density); as a measure of colony strength, these metrics are closely related to colony weight/productivity, colony size, and reproductive capabilities (Chabert et al., 2021; Taha & Naser Al‐Kahtani, 2013). To measure worker density without disturbing the workers (without smoke), we photographed both sides of two frames per hive and calibrated the image size using a five‐square‐centimeter grid. Then, the worker bees were removed with a brush, and the immature worker density was photographed on both sides. We used ImageJ software (Abramoff et al., 2004) to manually count workers and broods from the photographs.

In Mexico, beekeepers generally use two types of technical hives, Jumbo and Langstroth that differ in the volume of the brood chamber (Carlos & Castellanos, 2020). In our sampling, it was observed that 56% of the hives corresponded to the Langstroth type, while 44% were of the Jumbo type. To standardize colony sizes and make hives comparable throughout the experiment, we calculated bee density by dividing total worker counts by the total area covered by the frames in ImageJ (Delaplane et al., 2013).

### 2.2 Pollen sampling and preparation

To ensure that the collected pollen accurately reflects the environmental conditions, specifically the floral resources closest to the collection date, we randomly selected only those cells containing freshly collected pollen, which differed in appearance from pollen stored for several weeks. Pollen was gathered from both brood frames and reserve frames, employing a sterile stainless‐steel spatula for gentle scraping from the cells. In our study, we maintained a consistent amount of pollen collected for each hive. To achieve this uniformity, we utilized 8‐cubic centimeter tubes that had been pre‐loaded with 5 cubic centimeters of 95% molecular‐grade alcohol. These tubes were filled (30–50 cells) with pollen sourced from the hive chosen for sampling and then stored at −80°C in the laboratory. For further analyses, the samples were dried at 56°C for 24 h, weighed, and then re‐suspended in a 1:3 ratio of 1× phosphate‐buffered saline solution. The samples were homogenized for 5 min in a shaker, and an aliquot of 100 μL (27.7, ± SD 0.8 mg of pollen, *n* = 30) was taken for DNA extraction. Pollen subsamples were re‐suspended in 400 μL buffer Cell Lysis Solution from the Promega DNA isolation kit and then mechanically disrupted in a Bead‐Blaster homogenizer (Benchmark) for 4 min at 7 m/s with 100 mg of 3.0 mm glass beads (Sigma‐Aldrich). The Promega DNA isolation kit was used to extract DNA from the pollen samples. To avoid PCR bias, separate libraries for ITS2 and trnL were prepared in 25 μL reactions. Universal primer sets (Table S1), previously employed in pollen metabarcoding, were utilized for the amplification, resulting in amplicon lengths of approximately 539 and 328 bp for ITS2 and trnL, respectively (Richardson et al., 2018; Sickel et al., 2015). This selection of primer sets is based on their successful application in previous studies and takes into account the availability of sequences in public databases (Milla et al., 2021). Negative and positive amplification and DNA extraction controls for both markers were included to check for contaminants in the laboratory workflow. Equal concentrations of both libraries were pooled and sequenced. An Illumina MiSeq platform using 2 × 250 cycles V2 chemistry (Illumina, San Diego, CA, USA) was used with a dual‐index inline barcoding method to prepare metagenomic libraries for sequencing. The sequencing was carried out by LANASE‐UNAM genomic services.

### 2.3 Reference database construction

We used RESCRIPt (Robeson et al., 2020) to generate a QIIME2‐compatible trnL and ITS barcoding reference database based on the Genbank genetic resources. On January 2021, we downloaded all available phanerogam plants trnL and ITS sequences from NCBI using two searches: txid33090 [ORGN] AND (trnL‐trnF intergenic spacer [Title] OR trnL [Title] OR trnT‐trnL [Title] OR tRNA‐Phe (trnF) gene [Title] OR tRNA‐Leu (trnL) gene [Title]) for trnL, and txid33090[ORGN] AND (internal transcribed spacer ITS1 [Title] OR ITS1 [Title] OR internal transcribed spacer 1 [Title] OR internal transcribed spacer ITS2 [Title] OR ITS2 [Title] OR internal transcribed spacer 2 [Title]) for ITS. We removed sequences with less than five ambiguous bases and/or any homopolymer longer than 12 bp, and those that were either too short or too long for the barcode region of each marker. To combine the UNITE, NCBI, and PLANiTS databases, we exported the remaining sequences and taxonomies from QIIME2 (Banchi et al., 2020), dereplicated them to only include identical sequence records with distinct taxonomy and, by using the CONABIO floristic checklist (http://www.conabio.gob.mx/informacion/gis/), geographically restricted DNA barcoding reference databases to Mexico. The trnL database contained 21,760 plant sequences comprising 58 orders, 238 families, 2014 genera, and 6541 species. The ITS database contained 13,080 plant sequences, including 60 orders, 250 families, 2491 genera, and 11,591 species. This database collectively represents around 36% and 20% of the flowering plants (native, exotic, and invasive species) present in Mexico (CONABIO, 2021).

### 2.4 Pollen analysis

Output reads were directly obtained from the Illumina MiSeq sequencer, which included demultiplexed fastq files processed through the QIIME2 plugin (Bolyen et al., 2019). Quality check filtering, sorting of markers, denoising, forward and reverse read truncation, merging reads, and dereplication were all conducted using the replicable and open‐source QIIME2‐DADA2 plugin (Callahan et al., 2016). The pipeline was adapted to each marker according to their characteristics. The taxonomic allocation of amplicon sequence variants (ASVs) was determined through a global search within VSEARCH and a threshold of 96% similarity to delimit species against each local reference database in the qiime2‐feature‐classifier (Bokulich et al., 2018). Sequences assigned to hybrid varieties or subspecies were manually checked and reassigned to the next highest taxonomic rank, and all ASVs that were not assigned to vascular plant families were excluded from further analysis. All bioinformatics processing was conducted on the computer cluster of the LANASE‐UNAM. We imported plant ASVs into R language version 3.6.0 and manipulated them using the phyloseq statistical package (McMurdie & Holmes, 2013). To eliminate the possibility of cross‐contamination between samples, we excluded taxa with relative abundances lower than 0.01% (the average relative abundances in negative control samples). After quality control, 286 samples were used to determine the taxonomic identity of the pollen diet. We used the proportional abundances of each of the ITS2 different sequences in each sample to conduct ecological inferences. This approach aligns with recent studies highlighting a robust correlation between the quantity of ITS2 reads and the pollen levels estimated through microscopy and palynological techniques (Bänsch et al., 2020; Richardson et al., 2021; Wilson et al., 2021).

### 2.5 Landscape composition

To characterize the landscape structure of each study site, a supervised classification was made in QGIS from the multispectral satellite images SENTINEL 2 closest to the sampling date (±10 days of sensing frequency). Landscape composition was measured in terms of total cover for each land‐use class using the QGIS plugin LecoS (Jung, 2016) for calculating patch‐based landscape metrics within a radius of 2.5 km around the apiaries. The influence of the surrounding landscape was restricted to two buffer distances: the 2.5 km, which is considered the typical foraging distance for honey bees in agricultural areas (Danner et al., 2014). Additionally, the Shannon Index (SH), a measure of landscape elements and their proportional changes, was calculated. This metric of landscape diversity is independent of specific habitat types and is widely used in ecological studies (Danner et al., 2017). Moreover, we incorporated the forest cover types from Mexico's land use maps as covariables (Instituto Nacional de Estadística y Geografía, 2019).

### 2.6 Bioclimatic variables

Bioclimatic parameters were obtained from the National Network of Automated Agrometeorological Stations (LNMySR, 2021) and the National System of Meteorological Stations (EMA's, 2022). Meteorological data corresponding to the collection date at the apiary was extracted from the nearest meteorological station (average distance 7.5 ± SD 5.7 km, *n* = 52). This dataset included minimum temperature, maximum temperature, precipitation, and relative humidity (RH).

### 2.7 Statistical analysis

To calculate the Shannon diversity index in each sample, we used the standardized proportional abundances of the ITS2 data set, employing the function phyloseq::estimate_richness. For the ordination of hive pollen sources, we used the phyloseq package to conduct a principal component analysis (PCA). The beta‐diversity variance (species composition) was partitioned between the effect of the habitat, season, type of hive, agricultural intensity, and year using a permutational analysis of variance (PERMANOVA) with the adonis function in the Vegan statistical package (Oksanen et al., 2013). We employed a distributional regression model within the GAMLSS (Generalized Additive Models for Location, Scale and Shape) package in R (Timmerman et al., 2021) to investigate the cumulative impact of the continuous variables landscape diversity, agricultural area, urban area, maximum temperature, minimum temperature, RH, precipitation, latitude and longitude, and the categorical variables vegetation and year on total worker density and pollen diversity (measured by Shannon diversity). GAMs were performed for each response variable, with separate models created for total bee density and pollen diversity. Then, the distribution of a model family was adjusted for all models using machine learning with the check_distribution function of package performance (Lüdecke et al., 2021). Our data were not normally distributed and included repeated measures over time, but a GAM generalized additive model was appropriate. All models include a random effect for apiary (Site) to deal with the non‐independence of samples within each site and overdispersion in the data. Then, a backward selection approach was used to construct GAM models, starting with the complete models for each response variable and eliminating explanatory variables if they did not explain a significant amount of the variance (α = .05), to arrive at a minimal adequate model (Zuur et al., 2009). Finally, the optimal model was selected by comparing all models using the R “performance” library (Figures S8 and S9). Upon the final examination of the models, we did not identify autocorrelated residuals in either of them. Therefore, there is no need to incorporate autocorrelation structures into the models.

## 3 RESULTS

### 3.1 Pollen metabarcoding

**FIGURE 2:**
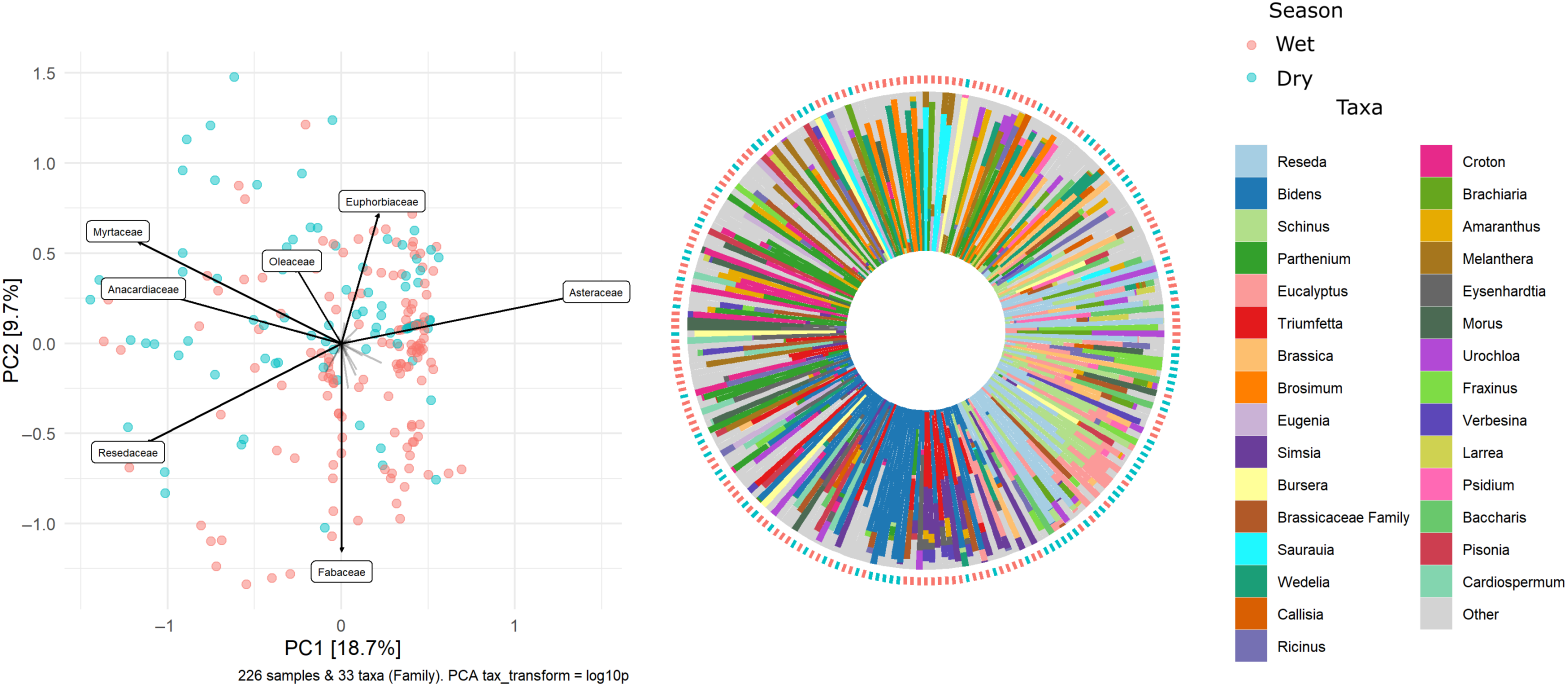
A principal component analysis (PCA) of family composition. The percentages shown on each axis indicate the contribution of the principal components to the total variance in the community composition. The iris plot shows the relative abundance patterns of 30 most abundant pollen species.

**FIGURE 3:**
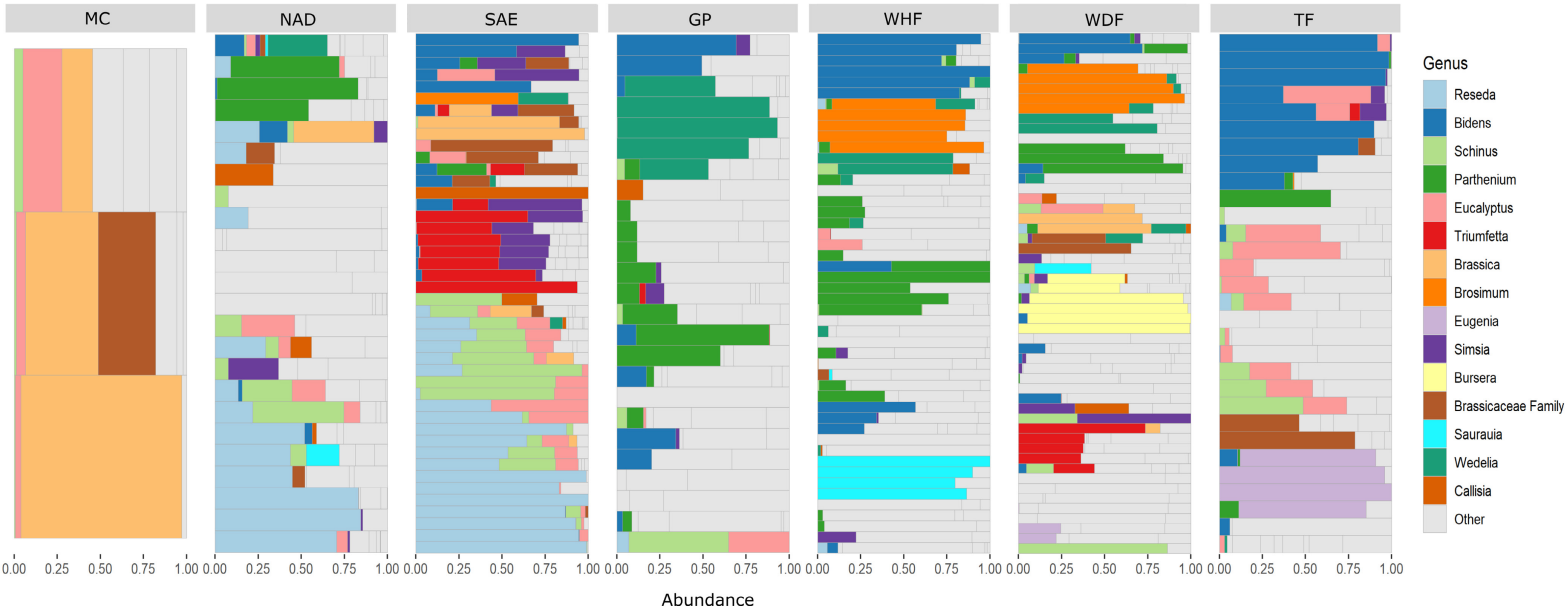
Relative abundance patterns of plant ASVs assigned to genus or other higher taxonomic categories detected in pollen samples collected by honeybees by region. The *y*‐axis represents the hives per ecoregion, and the term “Other” is used to denote taxa that do not fall within the top 15 most abundant categories. Ecoregions (MC = Mediterranean California, NAD = North American Deserts, SAE = Semiarid Elevations, TF = Temperate Forest, GP = Great Plains, WHF = Warm‐humid Forest, WDF = Warm‐dry Forest).

**FIGURE 4:**
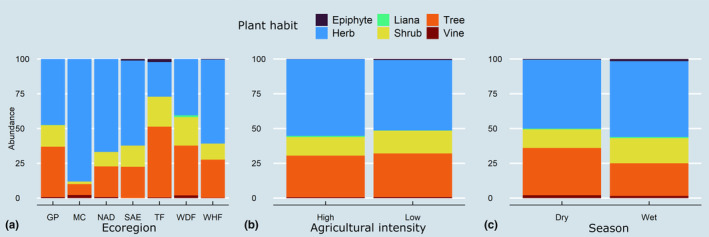
The proportion of the pollen of different plant habits in the samples classified by (a) ecoregion, (b) agricultural intensity, and (c) season. Ecoregions (MC = Mediterranean California, NAD = North American Deserts, SAE = Semiarid Elevations, TF = Temperate Forest, GP = Great Plains, WHF = Warm‐humid Forest, WDF = Warm‐dry Forest).

We obtained 1,020,577 filtered reads for ITS2 and 2,442,290 for trnL after implementing quality controls. According to the Mann–Whitney *U* test, we obtained significantly more sequences per sample using trnL (mean 7762) than ITS2 (mean 3239, *U* = 9.3, *p* = <.001, *n* = 612; Figure S1). Analysis of the 286 pollen samples identified 267 species from 243 genera and 80 plant families through the combined use of trnL and ITS2 barcode regions. Simultaneous identification of species utilizing both markers (ITS2 and trnL) was feasible for 43% of the families, 16% of the genera, and 4% of the species. This corresponded to a total of 35 families, 38 genera, and nine species for which concurrent identification was achieved (Figures S2–S4). On average, each sample (colony) contained 12 taxa, ranging from 1 to 34 plant species, with Asteraceae (46), Fabaceae (30), Euphorbiaceae (21), Myrtaceae (11), and Poaceae (10) being the most prevalent families (Figures S5 and S6). Notably, high levels of richness were observed in samples of pollen from tropical regions (WDF and WHF) with 124 and 101 plant genera, respectively. Arid and semiarid regions (regions GP, MC, NAD, and SAE) exhibited an average of 57 genera by region, while temperate regions (TF) contained 64 taxa. The PCA explains only 28.4% of the variation in the first two principal components. Despite this, the PCA analysis reveals that *A. mellifera* exhibited significant utilization of pollen from various floral resources, including Asteraceae, Resedaceae, Myrtaceae, Euphorbiaceae, Fabaceae, Anacardiaceae, and Oleaceae, across the sampled area (Figures 2 and 3). Ecoregions, seasons, and agricultural intensity all contributed to variations in growth/habit proportions of pollen sources, with herbs serving as the primary source, followed by trees and shrubs (Figure 4b,c). The results from the PERMANOVA analysis indicate significant differences among samples, with the site (apiary) emerging as the most influential variable in the model, contributing significantly with an *r* ^2^ value of .46 (Table 2).

**TABLE 2:** Variance partition of plant ASV community beta‐diversity in permutational multivariate analysis of variance with 9999 permutations.

	df	SS	*R* ^2^	*F*	Pr(>*F*)
Site	52	47.365	.45788	3.2656	**0.0001**
Year	2	1.717	.01659	3.077	**0.0001**
Season	1	0.609	.00589	2.1844	**0.0012**
Type of hive	1	0.104	.00101	0.3736	0.9913
Agricultural intensity	1	0.513	.00496	1.8401	**0.0004**
Residual	167	46.582	.4503		
Total	225	103.446	1		

*Note*: Significant *p* < .05 highlighted in bold.

Abbreviations: df, degrees of freedom; SS, sum of squares.

### 3.2 Landscape composition

Within a radius of 2.5 km around the apiaries, the landscape is composed of four major cover classes, as shown in Figure S7: natural vegetation, agricultural area, bare ground, and urban areas. The natural vegetation in the area is characterized by its notable diversity, with sampling that identifies 16 types of vegetation (Table 1). Among these, the Seasonal Dry Tropical Forest, the Medium Subperennial Forest, the Subtropical Shrubland, and the Pine and Oak Forest stand out, especially for their abundance in the beehive areas. Croplands are mostly made up of traditional (milpa) and extensive agriculture production, while intensive agriculture is the least encountered. The Agricultural area cover class varied from 0% to 86% of the total land area surrounding each apiary (Figure S7).

### 3.3 Influence of environmental and landscape factors on colony population size and pollen diet diversity

**FIGURE 5:**
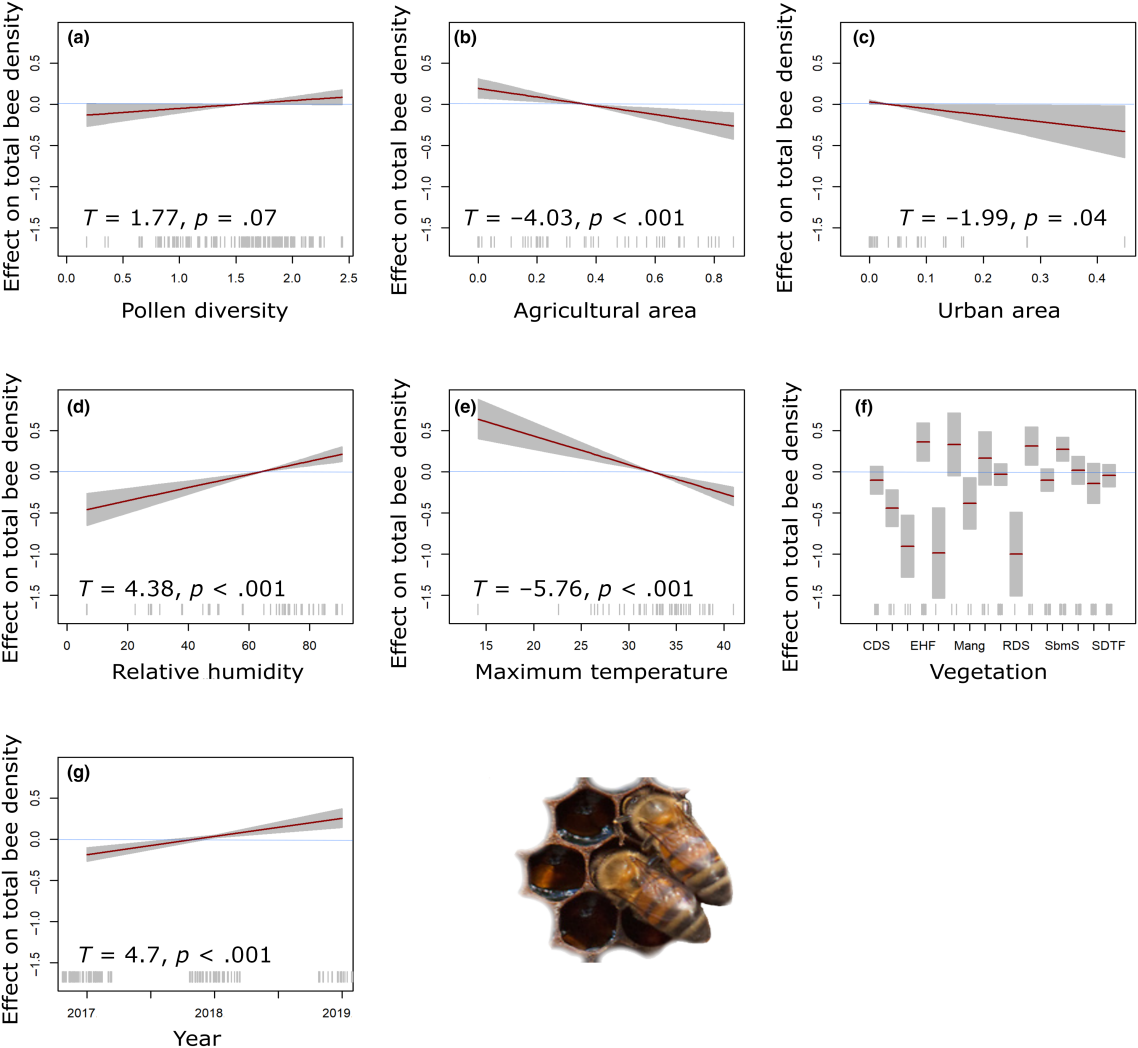
Estimated smoothness of, (a) pollen diversity, (b) agricultural area, (c) urban area, (d) relative humidity, (e) maximum temperature, (f) vegetation, and (g) year over the total bee density; the *y*‐axis is the partial effect of the variable and the shaded area represents 95% of the confidence interval around the main effect.

The diversity of pollen in the honey bee colonies and the density of bee colonies were significantly influenced by various factors within their apiary environment, including climate and landscape conditions. There were no statistically significant effects of pollen diversity on the total bee colony density (*T* = 1.71, *p* = .07; Figure 5a). The GAMs analysis revealed a negative correlation between total bee colony density and an increased agricultural area (*T* = −4.03, *p* < .001) (Figure 5b). Similarly, there was a negative correlation observed between total bee colony density and an increased urban area (*T* = −1.99, *p* = .04) (Figure 5c).

Furthermore, environmental variables played a significant role in determining total bee density. RH exhibited a positive relationship (*T* = 4.38, *p* < .001) (Figure 5d), while maximum temperature showed a negative correlation (*T* = −5.76, *p* < .001) (Figure 5e). The vegetation variable has a significant overall effect on total bee density (Figure 5f; Table 3). Results show a positive correlation between total bee density and the sampling year (*T* = 4.7, *p* < .001) (Figure 5g).

**TABLE 3:** Results of the GAMLSS models modeling the mean.

Model	Variable	Estimate	Std. error	*t* value	Pr(>|*t*|)	*R* ^2^
*Total bee density*						
	(Intercept)	−4.44E+02	1.69E−01	−4.751	<2e−16	
	pb(Shannon.ITS2.2.)	9.56E−02	5.38E−02	1.776	0.078305	
	pb(Agricultural.area)	−5.29E−01	1.31E−01	−4.031	9.83E−05	
	pb(Urban.area)	−8.01E−01	4.02E−01	−1.992	0.048685	
	pb(HR)	7.97E−03	1.82E−03	4.386	2.51E−05	
	pb(T..Max.)	−3.50E−02	6.07E−03	−5.762	6.66E−08	
	VegetationCF	−3.38E−01	1.46E−01	−2.313	0.02246	
	VegetationChap	−8.00E−01	1.84E−01	−4.351	2.88E−05	
	VegetationEHF	4.64E−01	1.38E−01	3.353	0.001071	
	VegetationGrass	−8.83E−01	2.72E−01	−3.25	0.001503	
	VegetationHV	4.35E−01	1.91E−01	2.272	0.024898	
	VegetationMang	−2.80E−01	1.70E−01	−1.651	0.101334	
	VegetationMDS	2.66E−01	1.62E−01	1.641	0.103499	
	VegetationPOF	6.98E−02	1.08E−01	0.644	0.520946	
	VegetationRDS	−8.96E−01	2.65E−01	−3.377	0.000989	
	VegetationSbdMF	4.14E−01	1.57E−01	2.64	0.009409	
	VegetationSbeMF	1.93E−03	1.21E−01	0.016	0.987315	
	VegetationSbmS	3.76E−01	1.14E−01	3.307	0.001246	
	VegetationSbS	1.21E−01	1.14E−01	1.064	0.289418	
	VegetationSDS	−3.89E−02	1.20E−01	−0.326	0.745238	
	VegetationSDTF	5.65E−02	1.21E−01	0.465	0.642527	
	Year	2.21E−01	8.38E−05	4.762	<2e−16	
Model	gamlss(formula = Total bee density ~ pb(Pollen diversity) + pb(Agricultural area) + pb(Urban area) + pb(Humidity relative) + pb(Maximum temperature) + Vegetation + Year + re(random = ~1|Site), family = WEI)					.24
*Pollen diversity*						
	(Intercept)	2.63E+02	1.96E−01	3.638	<2e−16	
	pb(Landscape.diversity)	2.88E−01	1.18E−01	2.442	0.016182	
	pb(Latitude)	−5.73E−02	1.53E−02	−3.737	2.94E−04	
	pb(HR)	3.81E−03	1.60E−03	2.377	0.019145	
	pb(Prec.)	−5.14E−04	3.80E−04	−1.354	1.79E−01	
	pb(T..Min.)	−1.44E−02	6.92E−03	−2.082	3.97E−02	
	pb(T..Max.)	1.84E−02	6.89E−03	2.664	0.008854	
	VegetationCF	−1.64E−01	1.32E−01	−1.247	2.15E−01	
	VegetationChap	9.12E−01	2.71E−01	3.368	0.001039	
	VegetationEHF	−5.03E−01	1.37E−01	−3.671	0.000371	
	VegetationGrass	5.05E−01	2.42E−01	2.091	0.038794	
	VegetationHV	8.37E−01	2.24E−01	3.734	0.000297	
	VegetationMang	3.36E−01	1.68E−01	1.994	0.048522	
	VegetationMDS	2.96E−01	1.57E−01	1.89	0.061364	
	VegetationPOF	−1.41E−01	9.33E−02	−1.506	0.134838	
	VegetationRDS	1.15E−01	2.34E−01	0.493	0.622921	
	VegetationSbdMF	−1.56E−01	1.43E−01	−1.088	0.278847	
	VegetationSbeMF	−3.00E−01	1.22E−01	−2.456	0.015592	
	VegetationSbmS	1.68E−01	1.09E−01	1.538	0.126916	
	VegetationSbS	3.77E−02	9.69E−02	0.389	0.698162	
	VegetationSDS	4.79E−01	1.25E−01	3.849	0.000197	
	VegetationSDTF	−3.54E−02	1.12E−01	−0.317	0.751602	
	Year	−1.30E−01	9.74E−05	−3.611	<2e−16	
Model	gamlss(formula = Pollen diversity ~ pb(Landscape diversity) + pb(Latitude) + pb(Humidity relative) + pb(Precipitation) + pb(Minimum temperature.) + pb(Maximum temperature) + Vegetation + Year + re(random = ~1|Site), family = WEI)					.35

*Note*: Vegetation (SbdMF = Sub‐deciduous Medium Forest, SbS = Subtropical Scrub, SbmS = Submontane Scrub, EHF = Evergreen High Forest, CDS = Crassicaule Desert Scrub, SDTF = Seasonally Dry Tropical Forest, Mang = Mangrove, SbeMF = Sub‐evergreen Medium Forest, SDS = Sarcocaule Desert Scrub, MDS = Microphyll Desert Scrub, HV = Halophilic vegetation, CF = Cloud Forest, Chap = Chaparral, RDS = Rosetophyllous Desert Scrub, POF = Pine‐Oak Forest, Grass = Grassland). Continuous predictor variables are specified within the model as (pb).

**FIGURE 6:**
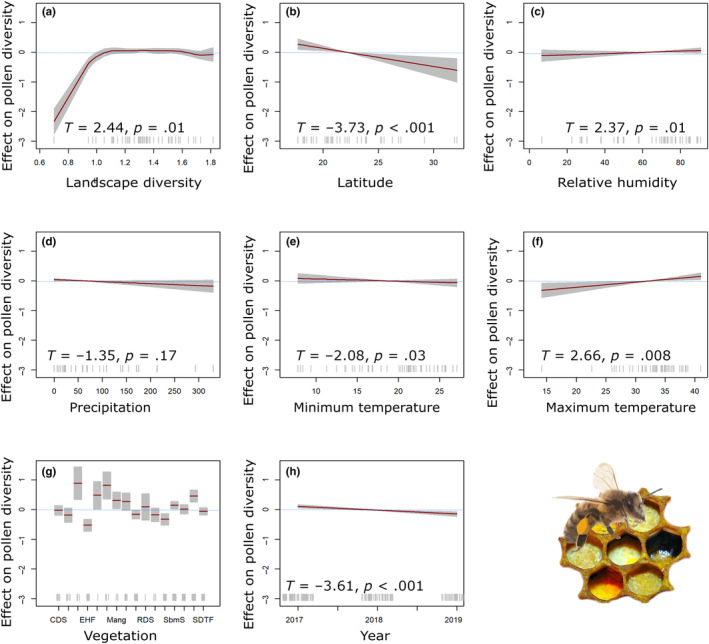
Estimated smoothness of, (a) landscape diversity, (b) latitude, (c) relative humidity, (d) precipitation, (e) minimum temperature, (f) maximum temperature, (g) vegetation, and (h) year over pollen diversity; the *y*‐axis is the partial effect of the variable and the shaded area represents 95% of the confidence interval around the main effect.

Analysis of GAMs revealed a significant positive correlation between pollen diet diversity and landscape diversity (*T* = 2.44, *p* = .01) (Figure 6a). Latitude has a significant negative impact on pollen richness (*T* = −3.73, *p* < .001) (Figure 6b). Similar to colony size, environmental predictors played a crucial role in influencing the diversity of pollen within the colonies. RH exerts a significant positive effect on pollen diversity (*T* = 2.37, *p* = .01) (Figure 6c); however, the variable precipitation does not appear to have a significant effect on pollen diversity (*T* = ‐1.35, *p* = .17) (Figure 6d). The results also indicate that ambient temperature around the apiary impacts pollen diversity. Minimum temperature demonstrates a negative marginal significant impact (*T* = −2.08, *p* = .03) (Figure 6e), while maximum temperature has a significant positive effect on pollen diversity (*T* = 2.66, *p* = .008) (Figure 6f). The vegetation variable has a significant overall effect on pollen diversity (Figure 6g; Table 3). Finally, a negative correlation was observed between pollen diversity and the sampling year (*T* = −3.61, *p* < .001) (Figure 6h).

## 4 DISCUSSION

Mexico is among one of the megadiverse countries on the planet, with an exceptionally high diversity of species used by humans (approximately 400 plant species), of which 150 are dependent on pollinators for fruit and seed production (Ashworth et al., 2009). Our study represents a pioneering investigation into *Apis mellifera*, a key pollinator of crops, across varying levels of agricultural or urban land use. We conducted a detailed investigation encompassing 52 apiaries located throughout Mexico, analyzing the quantitative aspects of each apiary habitat and the floral resources utilized by the bees. Our findings reveal that high levels of landscape diversity correspond with high pollen diet diversity. Our results indicate that when the agricultural area comprises more than 40% of the landscape surrounding the apiary, it negatively impacts the colony population. Our research underscores the need to promote biodiverse agricultural landscapes, preserve habitats, and safeguard natural spaces for bee well‐being and sustainable pollination services.

Our methodology encompassed DNA metabarcoding, a technique enabling precise taxonomic identification of plants used in the *A. mellifera* diet. We found 267 species, 243 genera, and 80 plant families, revealing a diverse foraging ability by honey bees in Mexico from different plant families like Asteraceae, Fabaceae, Poaceae, and Euphorbiaceae. These are the most prominent families in the Mexican flora (Villaseñor & Villaseñor, 2016). Despite the diversity of floral resources supporting Mexican honey production, an imbalance exists in the documentation of melliferous and polliniferous flora, with a stronger focus on the South rather than the North of the country (SADER, 2021). In that sense, our research offers valuable insights into the pollen preferences of bees in Mexico, that can help beekeepers manage their apiaries more effectively, by allowing them to take better advantage of polliniferous floral resources (Appendix S2). We show that distinct patterns of pollen preferences emerged across different apiaries, and diet diversity increased from the North to the South of Mexico. In arid and semiarid regions (MC, NAD, and SAE), *Reseda, Schinus, Parthenium, Triumfetta, Simsia, Croton, Larrea, Wedelia, Eysenhardtia, Morus, Verbesina, Amaranthus*, and *Brassica* show dominance. In temperate regions (TF), *Bidens, Baccharis, Fraxinus, Eucalyptus, Eugenia, Simsia, Ricinus*, and *Psidium* prevailed. Meanwhile, in tropical regions (WDF and WHF), there is dominance by *Bidens, Brosimium, Bursera, Pisonia, Melanthera, Saurauia, Eysenhardtia, Cardiospermum, Triumfetta, Brachiaria, Croton, Urochloa*, and *Parthenium* (Figure 3). Our DNA metabarcoding study shows that specific regions of the country host distinct plant community composition, including endemic flowering plants (Appendix S2). These findings could be used to confirm the origin of nectar and honey. This approach is important not just for understanding plant communities, and honey bee foraging preferences but also has practical implications in food authentication. Identifying unique bioactive properties infused into honey from its floral origin often adds significant value and increases its market appeal. This method has proven effective in determining both the floral and geographic sources of honey across different geographical scales (Khansaritoreh et al., 2020; Pathiraja et al., 2023).

We established a significant positive nonlinear correlation between pollen diet diversity and landscape diversity (Figure 6a). This finding is crucial for honey bee colony health, as floral diversity, abundance, and community composition directly influence food availability and nutritional content (Pasquale et al., 2013). Ensuring the availability of both abundant and high‐quality floral resources is critical for maintaining colony growth, as a decline in pollen supply may result in smaller colonies and fewer forager bees (Hass et al., 2019; Requier et al., 2017; Smart et al., 2019). Given its eusocial nature and ability to thrive in large colonies, *A. mellifera* relies heavily on ample floral resources and wide foraging areas to sustain its population (Kendall et al., 2022). Our results also show that increasing pollen diversity positively correlates with total bee density (Figure 5a). However, it is important to note that while these observed patterns lack statistically significant associations through GAMs models, the possibility of a diverse pollen diet impacting colony size remains plausible.

Regarding land use, increasing agricultural areas and urban areas negatively correlates with total bee density (Figure 5b,c). Habitat loss, a consequence of agricultural intensification and urbanization, emerges as a significant driver of pollinator decline. Agricultural intensification negatively impacts plant communities, consequently diminishing the availability of essential floral resources for honey bees and the abundance of pollinator species (Tommasi et al., 2021; Winfree et al., 2009). Pollen and nectar supply the energy and nutrients essential for honey bee colonies; hence, access to a diverse range of floral plant sources is crucial for the long‐term survival of colonies. Monocultures often offer floral resources for only brief periods, leading to nutritional stress among honey bees, potentially reducing immunocompetence and increasing parasite loads (Alaux et al., 2010; Dolezal et al., 2016a).

In addition to the limitations imposed by reduced floral resources and habitat size, agricultural intensification poses another significant threat through agrochemical exposure. Pollinators in agricultural landscapes can inadvertently transport pesticide‐contaminated pollen back to their hives, posing risks to their health (Dolezal et al., 2016b; Shanahan, 2022). Furthermore, herbicides employed in agricultural practices eliminate vital weed species that would otherwise be valuable floral resources for bees. Moreover, these herbicides may have both lethal and sublethal effects on honey bee populations (Requier et al., 2015).

On the other hand, urbanization represents one of the most intensive and rapidly expanding forms of landscape alteration (UNHSP, 2022). This process involves a profound restructuring of the landscape, leading to the loss and fragmentation of natural areas (Liu et al., 2016). Such transformations have far‐reaching consequences for biodiversity, thereby affecting ecosystem health and functionality (Casanelles‐Abella et al., 2022; Samuelson et al., 2022).

Given that plant diversity is shaped by ecosystem processes operating across various spatiotemporal scales, the pollen diversity inside colonies also exhibits spatial sensitivity. As a direct influencer of plant diversity, latitude emerges as a key geographical characteristic. Our research reveals a negative correlation between pollen diversity in the *A. mellifera* diet and latitude (Figure 6b). Supporting our findings, existing literature suggests a trend where plant diversity tends to rise with decreasing latitude in comparable habitats and elevations (Gentry, 1982).

Concerning climatic conditions, our study found a positive correlation between RH, and bee colony density (Figure 5d), which is likely due to the critical role of humidity in colony growth. Excessive humidity above 87% negatively affects brood survival due to raised microbial activity, while desiccation of honey bee eggs below 50% nest humidity reduces emerging larvae numbers (Ellis et al., 2008; Karbassioon et al., 2023). Like the relationship observed with total bee density, our study reveals a positive correlation between RH and pollen diversity (Figure 6c). This observation diverges from other studies where it was noted that the pollen‐collecting activity of bees diminishes with an increase in RH (Karbassioon et al., 2023). Our findings, however, emphasize that the observed patterns primarily underscore the substantial influence of humidity on the ecological conditions within the apiaries, subsequently impacting the diversity of plant species (Zhang et al., 2021).

On the other hand, our findings indicate a negative yet non‐significant relationship between precipitation and pollen diversity (Figure 6d). It is noteworthy, however, that other studies have reported a detrimental impact of precipitation on the foraging activity of *Bombus terrestris* (Peat & Goulson, 2005). The rationale behind this is that rain makes foraging more difficult and potentially dangerous. As a result, precipitation has a detrimental impact on flight activity, reducing pollen foraging.

Temperature is the most important predictor of honey bee activity (Clarke & Robert, 2018; Simioni et al., 2015). Similarly, our findings emphasize the importance of maximum temperature among other meteorological variables. Specifically, we observed a negative correlation, indicating that as temperatures rise, there is a corresponding decrease in the total bee density within the colony (Figure 5e). Previous experimental investigations suggest that bees disperse within the hive to avoid overheating at high temperatures, coupled with a decrease in the number of worker bees in the nest due to an increase in honey bee foraging activity with rising temperatures (Chabert et al., 2021; Karbassioon et al., 2023). Consequently, the rise in temperature negatively affects the number of bees inside the colony, leading to a decrease in total bee density. Foraging can take place in a wide temperature range, from 10 to 40°C, outside the hive (Abou‐Shaara, 2014); however, while both temperature variables exhibit significant effects (Figure 6e,f), maximum temperature is the best predictor. There is a greater predictive efficacy of maximum temperature in the GAMs model than with minimum temperature because of the broader thermal range of maximum temperature (14–42°C). The maximum temperature directly influences bee behavior, thereby influencing the availability of pollen resources in the hive through alterations in bee foraging activity. Our findings demonstrate a notable rise in pollen diversity corresponding to maximum temperature increases (Figure 6f).

The main threat to honey bees in the future is predicted to be climate change (Abou‐Shaara, 2016). Climate change can impact honey bee colonies negatively and/or positively, but in tropical regions, climate change has the potential to reduce honey yields by almost half (Delgado et al., 2012). Climate change models predict that parts of Mexico will experience more frequent droughts and a rise in temperature in the future (Estrada et al., 2022). Lack of rainfall would reduce plant bloom and survival, which would negatively impact honey yields but also reduce colony buildup and reproduction (Frazier et al., 2024). Indeed, recent long‐term data analysis has revealed a correlation between reductions in temperature range due to climate change and decreased honey yields in beekeeping production in Mexico (Balvino‐Olvera et al., 2023).

The vegetation variable has a considerable impact on total bee density and pollen diversity (Table 3), reflecting the diversity of ecosystems that they inhabit, and showing that not all geographical regions are equally favorable to commercial honey production. However, further investigation is necessary to quantify the ecological distinctions among honey bee colonies with varying degrees of European and African ancestry, particularly in the context of Mexico. Mexican honey bee colonies are primarily of African origin, yet the proportions of African lineage fluctuate based on factors such as management practices, beekeeping regions, and latitude (Quezada‐Euán, 2007). African honey bees are ecologically distinct in terms of reproduction, foraging, production, and behavior compared to bees from European honey bee stocks (Guzman‐Novoa et al., 2020).

On the other hand, the sample year has a considerable impact on total bee density and pollen diversity (Table 3). However, drawing qualitative conclusions regarding this variable is difficult due to the temporal restrictions of the study.

## 5 CONCLUSION

Our study of 52 apiaries across varying agricultural and urban landscapes reveals a strong link between landscape diversity and pollen diet diversity, underscoring the vital role of biodiversity in agricultural and urban ecosystems. Diverse plant species provide bees with abundant, high‐quality floral resources crucial for colony health and growth. The use of DNA metabarcoding has identified distinct pollen preferences across Mexico, offering practical insights for beekeepers to optimize apiary management. Furthermore, our research highlights the impact of climatic conditions on bee populations, emphasizing the need to integrate climate factors into beekeeping and land management strategies. The negative effects of agricultural intensification and urbanization on bee colony density emphasize the urgency of conserving habitats with diverse floral resources to ensure the well‐being of bee populations. In conclusion, promoting biodiversity, and safeguarding natural spaces are crucial for the long‐term sustainability of pollination services and beekeeping practices in Mexico. These findings stress the importance of targeted actions to protect and restore pollinator habitats and ensure access to diverse, high‐quality food resources for bee populations.

## AUTHOR CONTRIBUTIONS

**Francisco J. Balvino‐Olvera:** Conceptualization (equal); data curation (equal); formal analysis (equal); investigation (equal); methodology (equal); project administration (equal); software (equal); validation (equal); visualization (equal); writing – original draft (equal); writing – review and editing (equal). **Ulises Olivares‐Pinto:** Data curation (equal); formal analysis (equal); investigation (equal); methodology (equal); project administration (equal); software (equal); validation (equal); visualization (equal); writing – original draft (equal); writing – review and editing (equal). **Antonio González‐Rodríguez:** Conceptualization (equal); formal analysis (equal); funding acquisition (equal); investigation (equal); methodology (equal); project administration (equal); resources (equal); validation (equal); writing – original draft (equal); writing – review and editing (equal). **María J. Aguilar‐Aguilar:** Conceptualization (equal); data curation (equal); formal analysis (equal); investigation (equal); methodology (equal); writing – original draft (equal); writing – review and editing (equal). **Gloria Ruiz‐Guzmán:** Conceptualization (equal); data curation (equal); formal analysis (equal); investigation (equal); methodology (equal); validation (equal); writing – original draft (equal); writing – review and editing (equal). **Jorge Lobo‐Segura:** Conceptualization (equal); data curation (equal); formal analysis (equal); funding acquisition (equal); investigation (equal); methodology (equal); project administration (equal); validation (equal); writing – original draft (equal); writing – review and editing (equal). **Jorge Cortés‐Flores:** Conceptualization (equal); formal analysis (equal); funding acquisition (equal); investigation (equal); methodology (equal); supervision (equal); validation (equal); writing – original draft (equal); writing – review and editing (equal). **E. Jacob Cristobal‐Perez:** Formal analysis (equal); investigation (equal); methodology (equal); validation (equal); visualization (equal); writing – original draft (equal); writing – review and editing (equal). **Silvana Martén‐Rodríguez:** Conceptualization (equal); formal analysis (equal); funding acquisition (equal); investigation (equal); methodology (equal); resources (equal); supervision (equal); validation (equal); writing – original draft (equal); writing – review and editing (equal). **Violeta Patiño‐Conde:** Data curation (equal); formal analysis (equal); investigation (equal); methodology (equal); resources (equal); supervision (equal); validation (equal); writing – original draft (equal); writing – review and editing (equal). **Mauricio Quesada:** Conceptualization (equal); formal analysis (equal); funding acquisition (equal); investigation (equal); methodology (equal); project administration (equal); supervision (equal); validation (equal); writing – original draft (equal); writing – review and editing (equal).

## CONFLICT OF INTEREST STATEMENT

The authors declare that they have no conflict of interest.

## Supporting information


Appendix S1

Appendix S2

## Data Availability

Data have been deposited in the NCBI Sequence Read Archive, which can be found at https://www.ncbi.nlm.nih.gov/sra (accession ID PRJNA875936). The pipelines developed for pollen metabarcoding on Qiime2 can be accessed using https://zenodo.org/records/11111144. All samples and data were obtained with the beekeeper's agreement and under their supervision. Biological sampling is done following SEMARNAT (Secretary of the Environment and Natural Resources) regulations and conducted under collecting licenses (numbers: FAUT‐0256‐2019 and FAUT‐0256‐2021).
