# Supplementary material for: Effects of floral resources on honey bee populations in Mexico: Using dietary metabarcoding to examine landscape quality in agroecosystems
Source: Ecol Evol. 2024 Jun 17;14(6):e11456. doi: 10.1002/ece3.11456 (PMC11183941; doi:10.1002/ece3.11456)
Supplement: Supplementary file 1 — Appendix S1 [file ECE3-14-e11456-s001.pdf]

Supplementary materials

Effects of floral resources on honey bee populations in Mexico: using dietary metabarcoding to examine landscape quality in agroecosystems

Francisco J. Balvino-Olvera[1,2], Ulises Olivares-Pinto[3], Antonio González-Rodríguez[4], María J. Aguilar-Aguilar[1], Gloria Ruiz-Guzmán[1], Jorge Lobo-Segura[4, 5], Jorge Cortés-Flores[1, 7], E. Jacob Cristobal-Perez[1], Silvana Martén-Rodríguez[1], Violeta Patiño-Conde[1] and, Mauricio Quesada[1,4]

[1] Laboratorio Nacional de Análisis y Síntesis Ecológica, Escuela Nacional de Estudios Superiores, Unidad Morelia, Morelia, Michoacán, 58190 México; [2] Posgrado en Ciencias Biológicas, Unidad de Posgrado, Edificio D, 1° Piso, Circuito de Posgrados, Ciudad Universitaria, Coyoacán, C.P. 04510, CDMX, México. [3] Escuela Nacional de Estudios Superiores Unidad Juriquilla, Universidad Nacional Autónoma de México, Boulevard Juriquilla 3001, Juriquilla, Querétaro, 76230 México; [4] Instituto de Investigaciones en Ecosistemas y Sustentabilidad, Universidad Nacional Autónoma de México, Morelia, Michoacán, 58190 México; [5] Universidad de Costa Rica, Escuela de Biología, San Pedro, 2600 Costa Rica; [6] Laboratorio Binacional de Análisis y Síntesis Ecológica, Escuela de Biología, Universidad de Costa Rica, San Pedro, 2600 Costa Rica; [7]Jardín Botánico, Instituto de Biología, Sede Tlaxcala, Universidad Nacional Autónoma de México, Santa Cruz Tlaxcala, México.

Correspondence

Mauricio Quesada

Email: mquesada@cieco.unam.mx

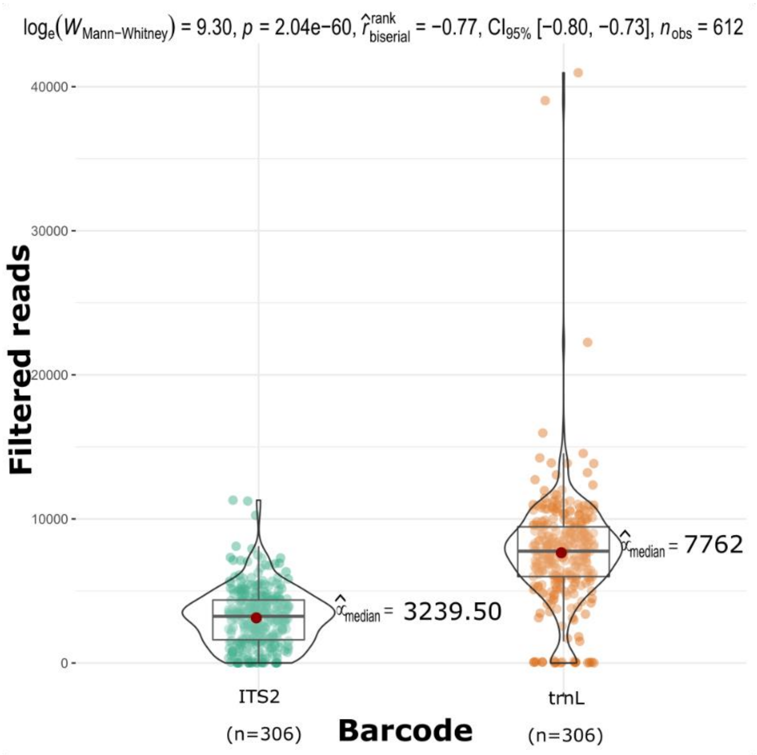

FIGURE S1. Number of reads post filtering per sample by different metabarcoding markers. The green color represents ITS2, while the orange color represents trnL.

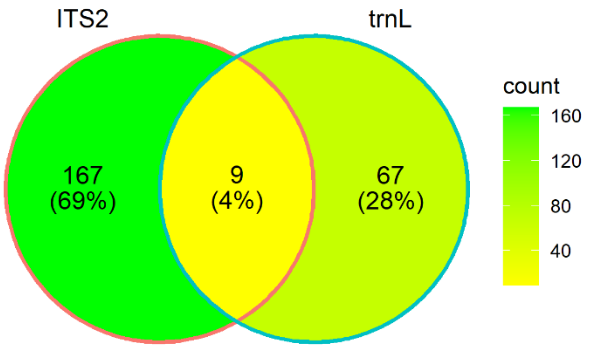

FIGURE S2. Venn diagrams illustrating the number of ASVs (Amplicon Sequence Variants) shared at the species level between ITS2 and trnL barcoding markers.

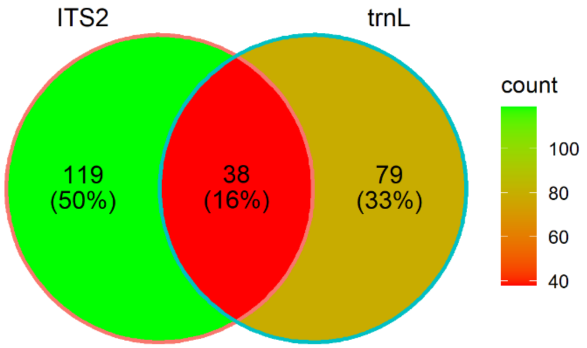

FIGURE S3. Venn diagrams illustrating the number of ASVs (Amplicon Sequence Variants) shared at the genus level between ITS2 and trnL barcoding markers.

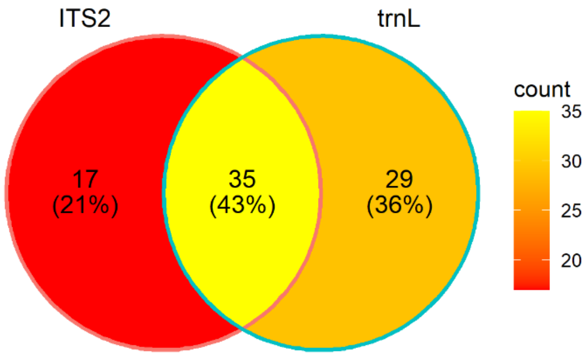

FIGURE S4. Venn diagrams illustrating the number of ASVs (Amplicon Sequence Variants) shared at the family level between ITS2 and trnL barcoding markers

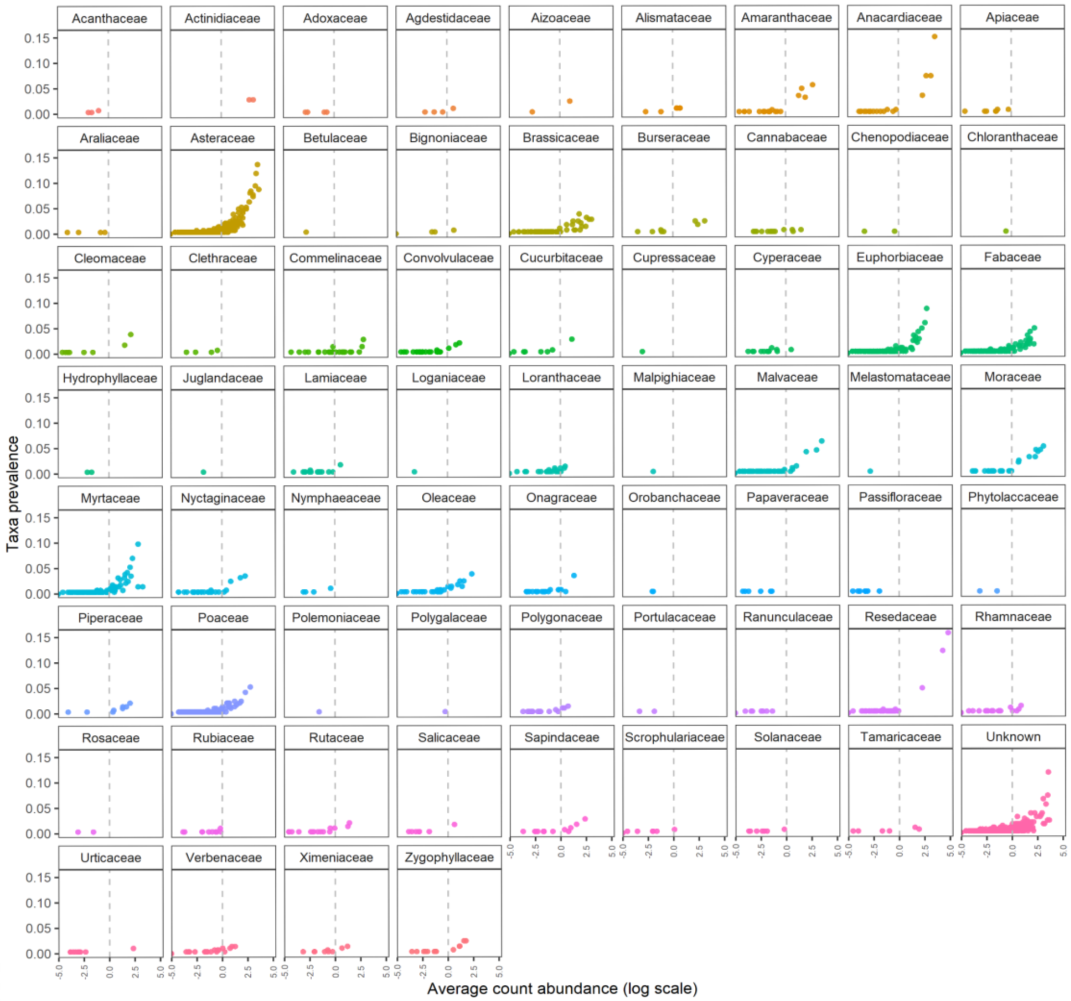

FIGURE S5. Prevalence plot (taxa prevalence vs average count abundance) for plant taxa representing the family level diversity across samples using ITS2 barcode. Each point corresponds to a different or unique taxon.

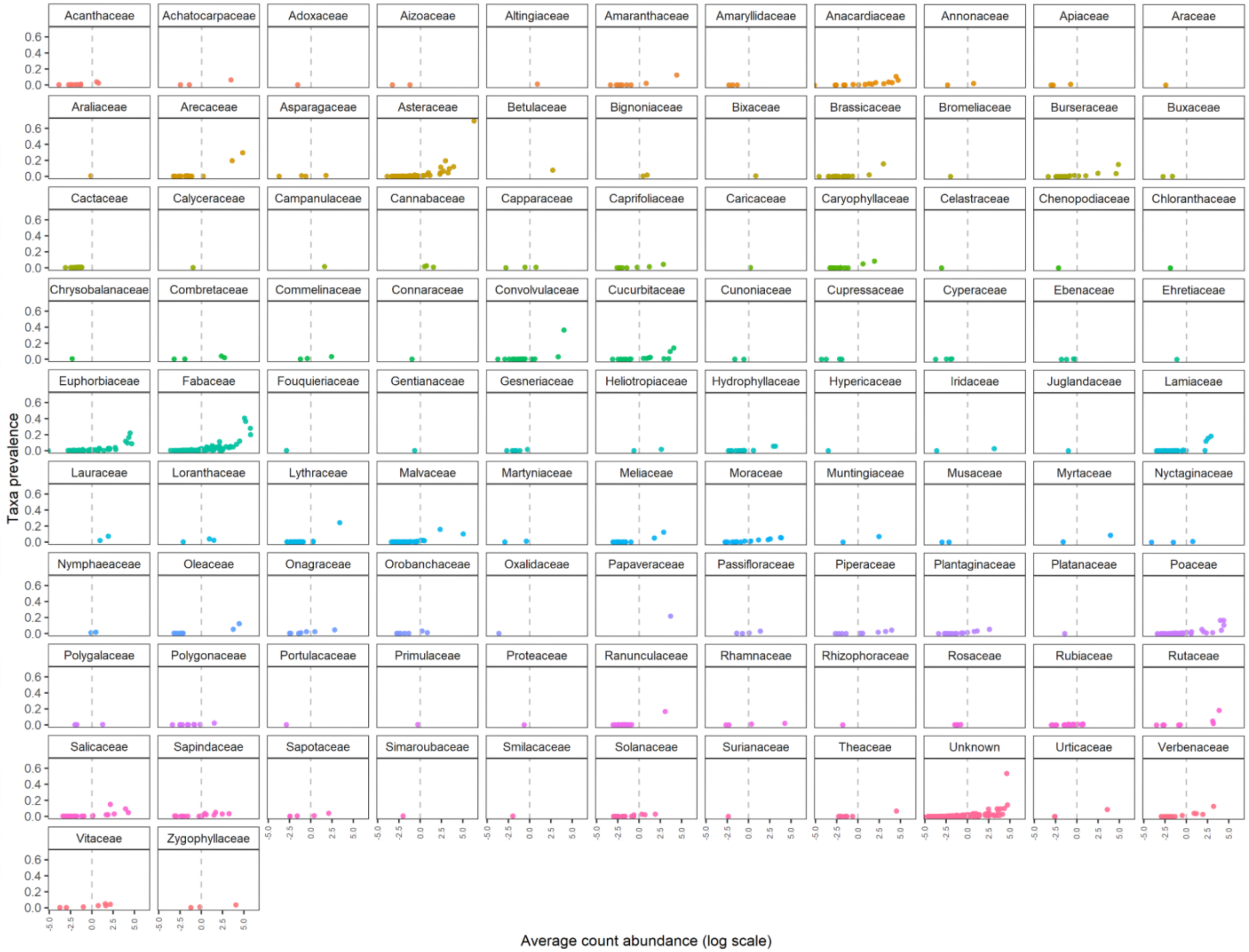

FIGURE S6. Prevalence plot (taxa prevalence vs average count abundance) for plant taxa representing the family level diversity across samples using trnL barcode. Each point corresponds to a different or unique taxon.

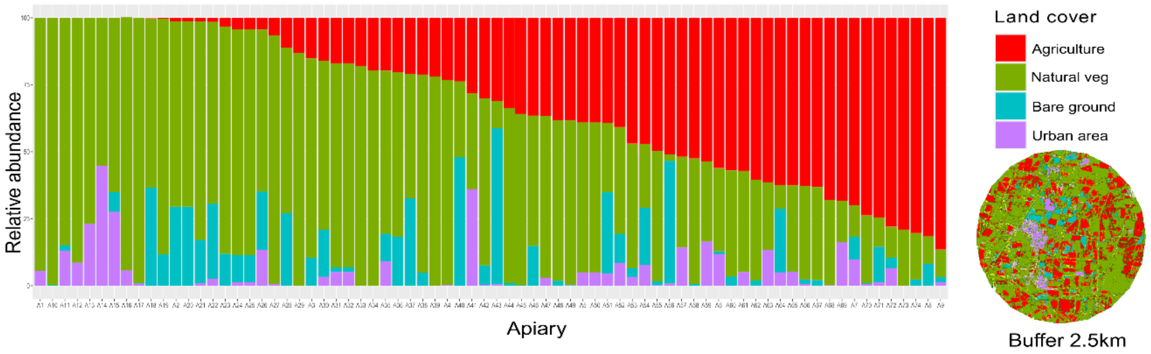

FIGURE S7. Composition of the landscape within a 2.5 km radius. Each bar in the graph represents a distinct apiary, while the agricultural area category encompasses all crop groups.

Model adjustment for total bee density

Initially, a preliminary analysis was conducted for each response variable (total bee density and pollen diversity), with all explanatory variables estimated in a complete model. Subsequently, a backward selection approach was adopted, starting from the complete models associated with each response variable. During this process, explanatory variables that did not contribute significantly to explaining the variance ($\alpha = 0.05$) were removed, aiming to reach a minimal yet adequate model (Zuur et al., 2009).

In a later stage, the optimal model was selected by comparing all models using the "performance" library in R.

```
library(gamlss)
wb0 <- gamlss(Bee.density ~
              pb(Shannon.ITS2.2.)+
              pb(Landscape.diversity) +
              pb(Latitude) +
              pb(Longitude) +
              pb(Agricultural.area) +
              pb(Urban.area) +
              pb(HR) +
              pb(Prec.) +
              pb(T..Min.)+
              pb(T..Max.)+
              Vegetation +
              Year +
              re(random = ~1|Site), data = datos, family=WEI)

wb2 <- gamlss(Bee.density ~
              pb(Shannon.ITS2.2.)+
              #pb(Landscape.diversity) +
              pb(Latitude) +
              pb(Longitude) +
              pb(Agricultural.area) +
              pb(Urban.area) +
              pb(HR) +
              pb(Prec.) +
              pb(T..Min.)+
              pb(T..Max.)+
              Vegetation +
              Year +
              re(random = ~1|Site), data = datos, family=WEI)

wb2.1 <- gamlss(Bee.density ~
```

```
              pb(Shannon.ITS2.2.)+
              #pb(Landscape.diversity) +
              pb(Latitude) +
              pb(Longitude) +
              pb(Agricultural.area) +
              pb(Urban.area) +
              pb(HR) +
              pb(Prec.) +
              #pb(T..Min.)+
              pb(T..Max.)+
              Vegetation +
              Year +
              re(random = ~1|Site), data = datos, family=WEI)

wb2.2 <- gamlss(Bee.density ~
              pb(Shannon.ITS2.2.)+
              #pb(Landscape.diversity) +
              pb(Latitude) +
              #pb(Longitude) +
              pb(Agricultural.area) +
              pb(Urban.area) +
              pb(HR) +
              pb(Prec.) +
              #pb(T..Min.)+
              pb(T..Max.)+
              Vegetation +
              Year +
              re(random = ~1|Site), data = datos, family=WEI)

wb2.3 <- gamlss(Bee.density ~
              pb(Shannon.ITS2.2.)+
              #pb(Landscape.diversity) +
              #pb(Latitude) +
              #pb(Longitude) +
              pb(Agricultural.area) +
              pb(Urban.area) +
              pb(HR) +
              pb(Prec.) +
              #pb(T..Min.)+
              pb(T..Max.)+
              Vegetation +
              Year +
              re(random = ~1|Site), data = datos, family=WEI)

wb2.4 <- gamlss(Bee.density ~
              pb(Shannon.ITS2.2.)+
              #pb(Landscape.diversity) +
              #pb(Latitude) +
              #pb(Longitude) +
```

```
                            pb(Agricultural.area) +
                            pb(Urban.area) +
                            pb(HR) +
                            #pb(Prec.) +
                            #pb(T..Min.)+
                            pb(T..Max.)+
                            Vegetation +
                            Year +
                            re(random = ~1|Site), data = datos,family=WEI)

library(performance)
plot(compare_performance(wb0,wb2,wb2.1,wb2.2,wb2.3,wb2.4, rank = TRUE))
```

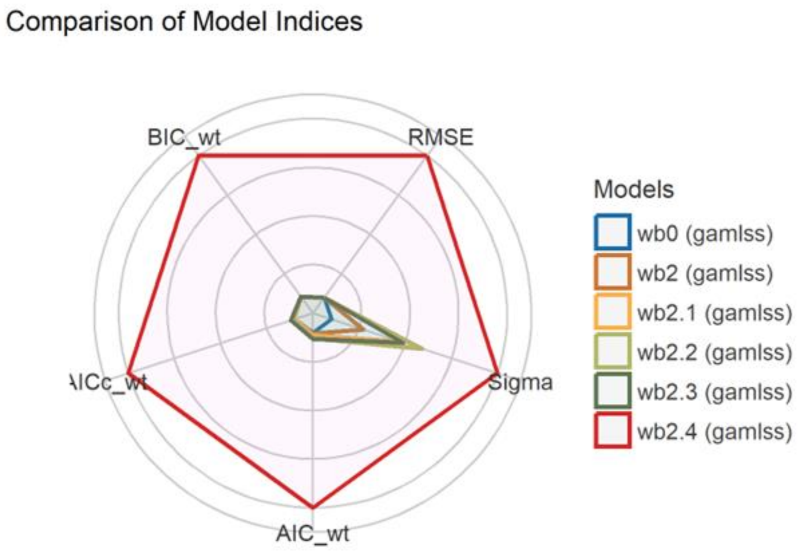

FIGURE S8. Comparison of indices for the response variable total bee density, starting from a complete model to the simplest model that only includes significant variables.

Checked the data for autocorrelation

```
library(performance)
check_autocorrelation(wb2.4)

OK: Residuals appear to be independent and not autocorrelated (p = 0.414).
```

The data are not autocorrelated, so there is no need to add autocorrelation structures to the model.

We calculated the deviance explained by the model.

```
# Calculate the deviance of the adjusted model
deviance_model <- deviance(wb2.4)

# Fit a null model (without covariates)
null_model <- gamlss(Bee.density ~ 1, data = datos, family = WEI)

# Calculate the deviance of the null model
deviance_null <- deviance(null_model)

# Calculate explained variation
explained_variance <- 1 - deviance_model / deviance_null

# Show explained variation
cat("Explained Variation:", explained_variance, "\n")

Explained Variation: 0.2432971

term.plot(wb2.4, pages = 1, ask = FALSE, rug = TRUE)
```

Model adjustment for total bee density

```
library(gamlss)
diet1 <- gamlss(Shannon.ITS2. ~
              pb(Landscape.diversity) +
              pb(Latitude) +
              pb(Longitude) +
              pb(Agricultural.area) +
              pb(Urban.area) +
              pb(HR) +
              pb(Prec.) +
              pb(T..Min.)+
              pb(T..Max.)+
              Vegetation +
              Year +
              re(random = ~1|Site), data = datos, family=WEI)

diet2 <- gamlss(Shannon.ITS2. ~
              pb(Landscape.diversity) +
              pb(Latitude) +
              pb(Longitude) +
              pb(Agricultural.area) +
              #pb(Urban.area) +
              pb(HR) +
              pb(Prec.) +
              pb(T..Min.)+
              pb(T..Max.)+
```

```
              Vegetation +
              Year +
              re(random = ~1|Site), data = datos, family=WEI)

diet3 <- gamlss(Shannon.ITS2. ~
              pb(Landscape.diversity) +
              pb(Latitude) +
              pb(Longitude) +
              #pb(Agricultural.area) +
              #pb(Urban.area) +
              pb(HR) +
              pb(Prec.) +
              pb(T..Min.)+
              pb(T..Max.)+
              Vegetation +
              Year +
              re(random = ~1|Site), data = datos, family=WEI)

diet4 <- gamlss(Shannon.ITS2. ~
              pb(Landscape.diversity) +
              pb(Latitude) +
              #pb(Longitude) +
              #pb(Agricultural.area) +
              #pb(Urban.area) +
              pb(HR) +
              pb(Prec.) +
              pb(T..Min.)+
              pb(T..Max.)+
              Vegetation +
              Year +
              re(random = ~1|Site), data = datos, family=WEI)

diet4 <- gamlss(Shannon.ITS2. ~
              pb(Landscape.diversity) +
              pb(Latitude) +
              #pb(Longitude) +
              #pb(Agricultural.area) +
              #pb(Urban.area) +
              pb(HR) +
              pb(Prec.) +
              pb(T..Min.)+
              pb(T..Max.)+
              Vegetation +
              Year +
              re(random = ~1|Site), data = datos,family=WEI)

diet5 <- gamlss(Shannon.ITS2. ~
              #pb(Landscape.diversity) +
              pb(Latitude) +
```

```
                    #pb(Longitude) +
                    #pb(Agricultural.area) +
                    #pb(Urban.area) +
                    pb(HR) +
                    pb(Prec.) +
                    pb(T..Min.)+
                    pb(T..Max.)+
                    Vegetation +
                    Year +
                    re(random = ~1|Site), data = datos, family=WEI)


library(performance)
plot(compare_performance(diet1,diet2,diet3,diet4,diet5, rank = TRUE))
```

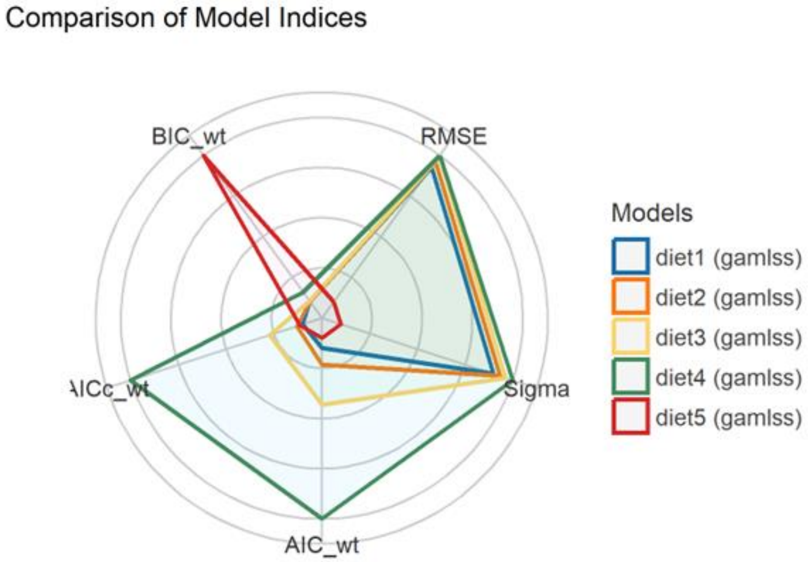

FIGURE S9. Comparison of indices for the response variable pollen diversity, starting from a complete model to the simplest model that only includes significant variables.

Checked the data for autocorrelation

```
library(performance)
check_autocorrelation(diet4)
```

```
OK: Residuals appear to be independent and not autocorrelated (p = 0.410).
```

The data are not autocorrelated, so there is no need to add autocorrelation structures to the model.

We calculated the deviance explained by the model.

```
# Calculate the deviance of the adjusted model
deviance_model <- deviance(diet4)

# Fit a null model (without covariates)
null_model <- gamlss(Shannon.ITS2.2. ~ 1, data = datos, family = WEI)

# Calculate the deviance of the null model
deviance_null <- deviance(null_model)

# Calculate explained variation
explained_variance <- 1 - deviance_model / deviance_null

# Show explained variation
cat("Explained Variation:", explained_variance, "\n")

Explained Variation: 0.3531346

term.plot(diet4, pages = 1, ask = FALSE, rug = TRUE)
```

Table S1. Primer sequences were used in this study. Illumina adapters are the sequences in black font.

**Primer**	**Sequence (5'- 3')**
trnL-C	CGAAATCGGTAGACGCTACG
	**TCGTCGGCAGCGTCAGATGTGTATAAGAGACAG**
trnL-H	CCATTGAGTCTCTGCACCTATC
	**GTCTCGTGGGCTCGGAGATGTGTATAAGAGACAG**
ITS-p3	YGACTCTCGGCAACGGATA
	**TCGTCGGCAGCGTCAGATGTGTATAAGAGACAG**
ITS-u4	RGTTTCTTTTCCTCCGCTTA
	**GTCTCGTGGGCTCGGAGATGTGTATAAGAGACAG**

Appendix S2. Plant metabarcoding taxonomy.

References for a floristic list of the pollen species used by *A. mellifera* in Mexico from the bibliography (Appendix S2)

1. S. Acosta-Castellanos, L. Quiroz-García, M. de la L. Arreguín-Sánchez, R. Fernández-Nava, Análisis polínico de tres muestras de miel de Zacatecas, México. *Polibotánica*, 179–191 (2011).

2. F. Araujo-Mondragón, R. Redonda-Martínez, F. Araujo-Mondragón, R. Redonda-Martínez, Flora melífera de la región centro-este del municipio de Pátzcuaro, Michoacán, México. *Acta botánica mexicana* (2019) https:/doi.org/10.21829/abm126.2019.1444 (March 13, 2022).

3. C. I. Córdova-Córdova, E. Ramirez-Arriaga, E. Martínez-Hernández, J. M. Zaldívar-Cruz, Caracterización botánica de miel de abeja (Apis mellifera L.) de cuatro regiones del estado de Tabasco, México, mediante técnicas melisopalinológicas. *Universidad y ciencia* **29**, 163–178 (2013).

4. R. González Sandoval, *et al.*, ANÁLISIS PALINOLÓGICO DE LOS RECURSOS FLORALES UTILIZADOS POR Apis mellifera L. (HYMENOPTERA:APIDAE) EN CUATRO MUNICIPIOS DEL ESTADO DE GUERRERO, MÉXICO. *Tropical and Subtropical Agroecosystems* **19** (2016).

5. M. González-Suárez, *et al.*, Diversity of melliferous flora in the State of Tamaulipas, Mexico. *Revista mexicana de ciencias pecuarias* **11**, 914–932 (2020).

6. B. Piedras Gutiérrez, D. L. Quiroz García, Estudio melisopalinológico de dos mieles de la porción sur del Valle de México. *Polibotánica* (2007) (December 11, 2018).

7. E. Ramírez-Arriaga, L. A. Navarro-Calvo, E. Díaz-Carbajal, Botanical characterization of Mexican honey from a subtropical region (Oaxaca) based on pollen analysis. *Grana* **50**, 40–54 (2011).

8. E. Ramírez-Arriaga, *et al.*, Análisis palinológico de mieles y cargas de polen de Apis mellifera(Apidae) de la región Centro y Norte del estado de Guerrero, México. *Botanical Sciences* **94**, 141–156 (2016).

9. F. Santana-Michel, Flora melífera del estadode Colima, México. *Boletín del Instituto de Botánica* **6**, 251–277 (2000).

10. V. Franco-Olivarez , M. Siqueiros-Delgado, E. Hernández Ayala. Flora apícola del estado de Aguascalientes. UAA, México (2012).

11. R. Riviera-Vázquez and A. Maldujano-Bueno. Flora nectarífera y polinífera de Guanajuato. Libro técnico Núm 7, campo experimental Celaya Guanajuato (2016).

12. G .Villegas Durán. Flora nectarífera y polinífera en el estado de Veracruz (2003).

13. G. Villegas., A. Bolaños., J.Miranda., & A. Zenón. Flora nectarífera y polinífera en el estado de Chiapas. Seceretaria de agricultura, ganaderia y desarrollo rural (2000).

14. G. Villegas., M. Bolaños., J. Miranda., J. García., & O. Galván. Flora nectarífera y polinífera en el estado de Tamaulipas. COTECOCA-SAGARPA, México, DF, 109. (2003).

15. G. Villegas., A. Bolaños., J. Miranda., I. Quintana., E. Guzmán., & J. Zavala. Flora nectarífera y polinífera en el estado de Michoacán. DF, México: Secretaría de Agricultura Ganadería y Desarrollo Rural (1999).

16. G. Villegas., M. Bolaños., J. Miranda., & Q. González. Flora nectarífera y polinífera en el estado de Guerreo. SAGARPA. México (2003).

17. G. Villegas-Durán., A. Rodríguez-Rodríguez., J. Miranda-Sánchez., & H. Córdova-Wade. Flora nectarífera y polinífera en el Estado de Tabasco. ISPROTAB. Tabasco, México (2004).
